# Prenatal stress increased γ2 GABAA receptor subunit gene expression in hippocampus and potentiated pentylenetetrazol-induced seizure in rats

**DOI:** 10.22038/ijbms.2020.39519.9371

**Published:** 2020-06

**Authors:** Morteza Bagheri, Ehsan Saboory, Mehrdad Nejatbakhsh, Shiva Roshan-Milani, Leila Derafshpour, Hojjat Sayyadi, Yousef Rasmi

**Affiliations:** 1 Cellular and Molecular Research Center, Urmia University of Medical Sciences, Urmia, Iran; 2 Zanjan Metabolic Diseases Research Center, Zanjan University of Medical Sciences, Zanjan, Iran; 3 Department of Physiology, School of Medicine, Urmia University of Medical Sciences, Urmia, Iran; 4 Neurophysiology Research Center, Urmia University of Medical Sciences, Urmia, Iran; 5 Department of Biostatistics, School of Medicine, Urmia University of Medical Sciences, Urmia, Iran

**Keywords:** GABAA receptor, Infant, Prenatal stress, Rat, Seizure

## Abstract

**Objective(s):**

Stress during pregnancy is able to bring extensive effects on neurobehavioral development in offspring. The GABAergic system plays a pivotal role in neuronal excitability, which can be affected by prenatal stress (PS). This study aimed to evaluate impact of the PS on γ2 subunit of gamma-aminobutyric acid A (GABAA) receptor gene expression in the hippocampus and seizure induced by pentylenetetrazol (PTZ) in developing rats.

**Materials and Methods:**

In this experimental study, female Wistar rats were exposed to restraint stress during gestation and their offspring were studied on postnatal days 14 and 21 (P14 and P21, respectively) for epileptic behaviors and γ2 GABAA receptor subunit gene expression. Quantitative real-time PCR was used for evaluating the γ2 GABAA receptor subunit gene expression in rat pups. Meanwhile, PTZ was injected into the pups, and seizure behaviors were recorded for 60 min.

**Results:**

The results showed that γ2 subunit mRNA expression significantly increased in the hippocampus of the stressed pups. The expression level of γ2 subunit was higher on P21 compared to that on P14 in both groups. Number of seizures with tonic–clonic features increased in pups of stressed group compared to the control group. Prenatal stress significantly caused an increase in the total score of seizure on P21.

**Conclusion:**

The effect of PS on seizure susceptibility is age-specific; the increased γ2 subunit level in the hippocampus might be, at least in part, the underlying mechanism for PS-induced augmentation of seizures in immature rats.

## Introduction

Factors in external milieu during gestation are thought to play a critical role in development of nervous system and its consequent tasks, as broad brain growth take place during this period (1, 2). Many studies have shown that prenatal stress (PS) can induce early and long lasting impacts on development of neural system. It has been reported that PS potentiates pilocarpine- and pentylenetetrazol (PTZ)-induced epileptic behaviors and raises vulnerability to seizures in rat pups (3-5). Stress and the hypothalamic–pituitary–adrenal (HPA) axis may play a critical role in the pathophysiology of epilepsy. Steroid hormones in brain regions are intimately associated with the HPA axis regulation and may be fundamental mediators in epilepsy pathophysiology (6). These hormones alter cellular activity in all hippocampal subfields in a region-specific way. Generally, these hormones promote excitatory activity, although neurosteroids like tetrahydrodeoxycorticosterone (THDOC) lead to enhanced tonic inhibition, particularly in the dentate gyrus (7). It is well-documented that stress increases glucocorticoid hormones, thereby potentiating excitotoxic damage in hippocampal GABAergic neurons (8). 

The type A receptor of gamma-aminobutyric acid A (GABAA) is recognized as a vital inhibitory receptors in the neural tissue. This receptor is a ligand-gated Cl^-^ channel that yields both phasic and tonic inhibition (9). This receptor can be considered as the target of numerous remedies (for instance sedative, hypnotic, anti-convulsive, anesthetic, and anti-anxiety drugs) for treatment of mental disorders (10). The GABAA receptor is involved in the control of neural excitability, anxiety, feeding and drinking behavior, circadian rhythms, cognition, learning and memory (11, 12). In addition, this receptor modifies some mental illness including epilepsy and depression (13). This receptor is a quaternary protein structure that consists of five protein subunits and possessed of more than 18 subtypes with 8 subunits (ɑ1- ɑ6, ß1-ß3, Y1-Y3, δ, ɛ, θ, π and 1-3Ƥ). In CNS, the most plentiful GABAA receptors are expected to have 2ɑ, 2ß and one Y/or δ subunits (14). These receptor components have various distribution patterns that cause many biological functions in diverse brain areas (13).

In the hippocampus, evidence has accumulated that GABAA receptors might be composed of a5bγ2 subunits. The γ2 subunit is found at high concentrations in neuronal structures of the hippocampus. It is concentrated in dendrites of the stratum radiatum CA1 to CA3, in the stratum lacunosum molecular and in the molecular layer of the dentate gyrus (15). This receptor plays an important role in the pathomechanism of epilepsy, which causes phasic inhibition of the GABAA receptor (13). Twelve to 48 hr after kainic-acid-induced seizures, the γ2 became markedly elevated in pyramidal neurons of the CA1 to CA3 and in the respective dendrites projecting to the stratum radiatum (15). Several studies have shown that stress can increase neurosteroids such as allopregnanolone. Neurosteroids often exhibit anticonvulsant effects. The main target of these compounds is the GABAA receptor, which probably affects itself through the effect on the GABAA receptor and changes in expression of the GABAA receptor subunit (16, 17). The influence of PS on the seizure induced by PTZ is well-known; nevertheless, the link between the PS with the γ2 subunit gene expression of GABAA receptor and convulsive behaviors caused by PTZ on immature brain is not clear. Thus, this study aimed to evaluate the impact of PS on gene expression of this receptor in the hippocampus and seizure behaviors induced by PTZ in immature rats.

## Materials and Methods

***Subject***

The Ethics Committee of the Urmia University of Medical Sciences, Urmia, Iran (IR.UMSU.rec.1395.229) evaluated and accepted this experimental study. All the procedures and protocols were consistent with the rules of the Helsinki Declaration (2008), as per imitated in the rules of the Ethics Committee of the Health Ministry, Iran. Wistar rats (200±10 g) were kept in standard cages (four rats per cage) at 20-24 ^°^C with a normal 12 hr daylight cycle (lights on at 7:00 AM). The rats had access to food and water *ad libitum*. At 12 weeks of age, the female and male rats were coupled. Copulation session started at 6:00 PM, and the rats were checked for vaginal plugs at next 8:00 AM. The female rat with positive copulation plug was transferred to another cage where 3 pregnant dams were housed for the whole pregnancy period. The females with no vaginal plug were returned to their home cage for a new coupling session. Then, the pregnant females were divided into two groups (n=6 each), namely as control and restraint stress groups. The rats in stress group were subjected to stressor (immobility in a restrainer) on days 15^th^, 16^th^, and 17^th^ of gestation (E15, E16, and E17, in that order); our previous studies showed that this period has remarkable effect on offspring (2, 18). 

***Restraint stress***

The immobility stress consists of transporting the pregnant rats from their cage to another room (experimental lab) as well as placing them in a restrainer (a transparent, plastic, cylindrical chamber with a six cm diameter and 16 cm length) under usual lab conditions. The rats were stressed for 2 hr (twice daily, 8-10 AM and 3-5 PM) for 3 successive days. The control rats (n=6, pregnant female) were treated similar to the stress rats, but without any intended stress.

***Collection of hippocampal tissue***

Following delivery, the mother and offspring were transferred to another cage and the litter size culled to eight for every single dam. Two pups per dam (one male and one female) at each indicated day (P14 and P21) were truncated under ether inhalation anesthesia. Then, the whole hippocampus was extracted rapidly and retained in standard saline ice. Tissue samples of hippocampus (51 mg each) were harvested and homogenized in 1 ml of Ribo EX solution, which is RNA protective (Gene all, South Korea). Then, the homogenate was incubated at room temperature for 5 min that allowed the complexes of nucleoprotein to entirely separate. At the end, the processed samples were kept at -80 ^°^C.

***Extraction of RNA and reverse transcription polymerase chain reaction (RT-PCR)***

Total RNA was extracted from the hippocampal samples by means of the Hybrid-R miRNA kit according to the manufacturer’s guidelines with slight adjustment (Gene all, South Korea). Then, the sample was suspended in the RNase free water and kept at -70 °C. RNA concentration was evaluated via determining the ratio of optical density at 260 nm and 280 nm and electrophoresis on agarose gel (1%). The cDNA was produced based on the RNA information of each sample (2 μl) by means of a commercial kit (Revert-Aid cDNA synthesis, Thermo-Fisher-Scientific, USA). The newly produced cDNA was directly applied as a pattern for RT-PCR by the succeeding reaction: 2 μl of cDNA template was amplified in 25 μl of total reaction volume containing 0.5 μl of each specific primer ß actin (housekeeping gene) and γ2 (target genes). We used two sets of primers as designed by Briner *et al* (19). Primer sequences for ß actin were 5´-cctggcacccagcacaat-3´ (sense) and 5´-gggccggactcgtcatact-3´ (anti-sense). In case of γ2, the sequences of primer were 5´- gtgaagacaacttctggtgactatgtggt-3´ (sense) and 5´- catattcttcatccctctcttgaaggtg-3´ (anti-sense). The amplification of PCR was performed by means of a Gene Amp 9600 PCR system (Perkin Elmer, Norwalk, CT, USA) in these circumstances: first denaturation at 94 ºC for three minutes followed by 30 augmentation rounds, each containing of denaturation at 94 ^°^C for 30 sec, annealing at 59 ^°^C for 30 sec and extension at 72 ^°^C for 50 sec, as well as an extra extension stage toward the end of the process at 72 ºC for five minutes. Then, the subsequent product was detached on agarose gel (2%) and stained by safe stain. Finally, the resultant gel was read under UV light and its band width was determined with a DNA Ladder of 100 bp.

***Real-time PCR***

The RT- PCR was performed by means of a ready to use kit of Maxima SYBR Green / ROX qPCR Master Mix (Thermo Fisher Scientific, USA) in a volume of 25 μl consistent with the instruction of manufacturer. Expression level of the aforementioned genes was examined by means of an iQ5 RT-PCR detection system (Bio-Rad, CA, USA). The experiment was arranged in an optical plate with 96-well lasting 10 min at 95 ^°^C followed by 40 rounds each lasted 15 sec at 95 ^°^C with 60 sec at 59 ºC. Measuring of the samples was performed twice. The relative expression level of the studied genes was calculated by the 2^-^^ΔΔCT^ method (20), standardized to beta-actin, and statistically evaluated for any possible significant difference (21). The findings are stated as mean±standard error (SE). 

***Behavioral assessment***

Different pups from each litter were used for behavioral studies in the experimental groups. On P14 and P21, the pups (both sexes equally, n=12 in each group at each day) were injected with the PTZ 45 mg/kg, IP. After the injection, the subjects were observed for epileptic seizures, and behavior of all pups was perceived and documented for 45 min by means of a digital camera. Seizure evaluation was performed by a formerly defined scale (3). In this scale, 0=no response, 1=ear and facial twitching, 2=myoclonic jerks without rearing, 3=myoclonic jerks with rearing, 4=turning over onto one side with tonic-clonic seizures and 5=turning onto back with generalized tonic-clonic convulsions. Additional factors including onset to first seizure and the number of tonic–clonic seizure were also examined. The similar procedure was performed on P21 for the rest of the rats (n=12, both sexes equally) in both groups. For each pup, seizure intensity was calculated using the following equation as total score of seizure (TSS):

TSS=SBS +1/LTCS *100 +NTCS +DTCS

Where SBS shows the sum-of behavioral-stages, LTCS stands for latency of tonic–clonic seizure (min), NTCS is the number of tonic–clonic seizures, and DTCS refers to the tonic–clonic seizures duration (min). Additional factors were also examined including onset of first change of behavior and onset of first highest convulsion (22, 23). P14 and P21 were chosen because our previous studies showed that they are appropriate and susceptible for assessing of PS-induced alterations in epileptic behaviors and molecular modifications (2, 5).

***Statistical analysis***

Normality of data distribution was checked using Kolmogorov–Smirnov test. Level of the mRNA expression between the groups was analyzed by the one-way analysis of variance (ANOVA) and Tukey’s test. Non-normally distributed data linked to epileptic behaviors were analyzed using Kruskal-Wallis test followed by the pair-wise evaluations. Statistical analyses were performed using SPSS (version 24, SPSS Inc, Chicago, IL, USA). Differences were considered to be significant at *P*<0.05.

## Results

The data of the pups for both sexes were separately analyzed. There was no significant difference between the male and female pups in terms of gene expression and epileptic behaviors. Therefore, the data of both sexes were combined and analyzed together.

***Effect of PS on gene expression of γ2 subunit***

**Figure 1:**
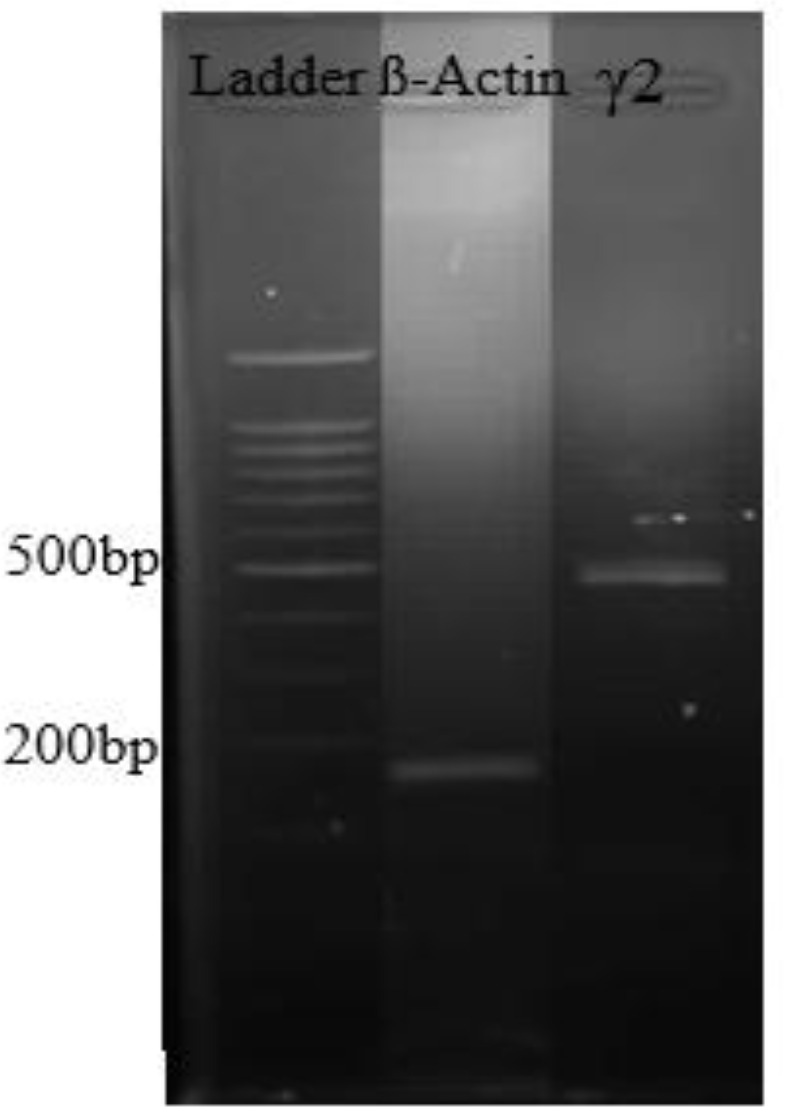
Predictable size of the RT-PCR results for the γ2 component of the gamma-aminobutyric acid (GABA) receptor and beta-actin in the immature rat; After PCR, the subsequent product was assessed on agarose gel (2%) and stained with safe staining. The stained gel was read under UV light and its band thickness was measured by a Ladder of DNA with 100 bp. The predictable sizes of the RT-PCR products were 144 bp and 460 bp for ß-actin and γ2 subunit, respectively

The estimated size of the RT-PCR products is presented in Figure 1. 

The findings of our study are summarized in Table 1. 

**Figure 2:**
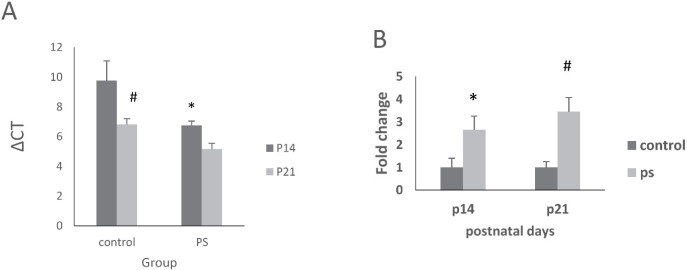
Evaluating the mRNA level of γ2 subunit by the quantitative real-time PCR in the control and stressed rats

Data analysis by one-way ANOVA revealed a remarkable difference among groups (F(3,20)=6.85, *P*=0.002, ANOVA). Further analysis by Tukey’s test indicated an important increase in the stressed group in comparison with the control group in terms of the hippocampal γ2 subunit gene expression on P14 and P21 (*P*=0.041 and *P*<0.05, respectively). The γ2 subunit gene expression on P21 was bigger than that on P14 in both groups (Figure 2).

***Influence of prenatal stress on seizure***

With the intention of comparing stress effects on seizure behaviors, statistical analyses was performed on data for epileptic behaviors (with non-normal distribution) by means of a Kruskal-Wallis ANOVA followed by multiple comparisons of all pair-wise evaluations. Time to onset of first epileptic behavior on P21 was significantly higher in the PS group than in the control group (H(3)=7.56, *P*<0.05). There was a non-significant decrease on onset of tonic clonic seizure and myoclonic jerk in the stressed pups compared to the control group on P14 and P21. Number of seizures with tonic-clonic features in the stressed pups was meaningfully (H(3)= 8.12, *P*˂0.05) greater than that in the control pups on P14. Duration of seizures with tonic-clonic features in the PS pups insignificantly increased on both days (Table 2). 

**Figure 3:**
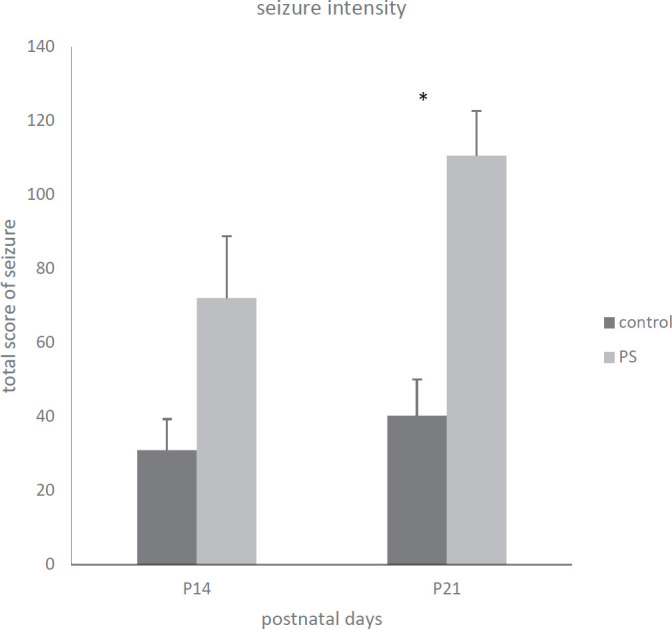
Consequence of stress during gestation on score of seizure induced by PTZ in immature rat (each bar n=12); total score of seizure (TSS) was designed and measured as intensity of seizure: TSS=SBS +1/LTCS *100+NTCS+DTCS (SBS=addition of behavioral stages, LTCS=latency of tonic–clonic seizure, NTCS=number of tonic–clonic seizures, and DTCS=duration of tonic–clonic seizures, P21: Postnatal day 21, PTZ: Pentylenetetrazol).* indicates *P<*0.05 with the control group on P21

The TSS was determined and considered as intensity of seizure. Moreover, the TSS meaningfully increased in prenatally stressed rats in comparison with the intact (control) rats on the both days, but it was statistically significant on P21 (H (3) =8.78, *P*<0.05, Figure 3).

**Table 1:** mRNA expression of the γ2 subunit of the GABAA receptor in the control and stressed rat pups

γ2 subunit mRNA expression		ΔCT±SEM	Fold ± SEM	*P*_value_
P14	PS control	6.75±0.39 9.75±1.33	2.65± 0.61 1± 0.40	0.041
P21	PS control	5.14±0.39 6.81±0.28	3.45± 0.62 1± 0.25	< 0.05

The rats were prenatally stressed and evaluated for PTZ-induced seizure at P14 or P21

GABAA: Gamma-aminobutyric acid A, PTZ: Pentylenetetrazol, PS: Prenatal stress, 14: Postnatal day 14, P21: Postnatal day 21

A: Assessing the value of ΔCT among the prenatally stressed (PS) and control rats on postnatal day 14 (P14) and 21 (P21); # indicates *P<*0.05 P14 vs P21 in the control group and * indicates *P=*0.04 P14 vs P21 in the stressed pups. B: Comparison of the fold change value among the stressed and control group on postnatal day 14 and 21. * indicates *P=*0.041 compared to the control; # indicates *P<*0.05 compared to the control. In general, there is an inverse relationship between ΔCT and fold change

**Table 2:** Impact of stress during gestation on PTZ-induced seizure in immature rat offspring

Epileptic behavior	Control		PS	
	P14 (n=12)	P21 (n=12)	P14 (n=12)	P21 (n=12)
Time to onset(s)	74.35±9.43	69.84±9.71	71.54±9.71	89.66±9.23*
latency to tonic clonic seizure(min)	29.99±5.34	36.43±4.27	25.86±6.28	24.35±5.83
Latency to myoclonic jerk(s)	14.32±3.50	8.99±2.65	7.39±2.57	3.88±1.29
Number of tonic-clonic	0.53±0.23	0.47±0.23	1.38±0.59*	1.42±0.35
Duration of tonic clonic(min)	10.79±7.72	70.53±23.42	85.92±1.68	10.79±1.58

Every single value represents the mean±SEM in the 12 rats

PS: Prenatal stress, P14: Postnatal day 14, P21: Postnatal day 21, PTZ: Pentylenetetrazol

* indicates significant difference with control on each relevant day (*P<*0.05)

## Discussion

In this study, γ2 subunit of the GABAA receptor gene expression in the hippocampus as well as seizure induced by PTZ was examined in rat pups that were exposed to stress on days 15-17 of their mothers’ pregnancy. Our findings indicated that mRNA level of γ2 component considerably augmented in the hippocampus on postnatal day 14 and 21 in stressed pups. Furthermore, γ2 component gene expression levels were bigger on both days in both groups (control and stressed pups). With respect to epileptic behavior, the PS increased the vulnerability to PTZ-induced seizures in pups and caused remarkable rise in number of the tonic-clonic seizure on P14. In addition, the total score of seizure in the stressed group was significantly higher on P21 compared to the unstressed animals.

The γ2 subunit of the GABAA receptor is located in different brain regions, including the hippocampal structures, cortex, thalamus and cerebellum, and it mainly participates in the formation of the GABAA receptor, which causes inhibition in the GABAA receptor and plays an important role in epilepsy (13, 24). The basic mechanism of the PS that induces a rise in the γ2 subunit in the hippocampal structures of rat is unknown. It seems that these changes are related to stress hormones and neurosteroids. The concentration of these compounds increases in response to stress; high level of neurosteroids can reach a value that, in addition to altering the activity of the GABAA receptor, may modify the mRNA levels of these subunits. These alterations in the GABAA receptor subunit expression following stress are likely mediated by neurosteroid-mediated effects on the GABAA receptor phosphorylation (25, 26). It has been reported that injection of allopregnanolone into the hippocampus leads to an increase in the expression of the γ2 subunit gene of the GABAA receptor (27). Similarly, Follesa *et al*. showed that encountering cerebellar granular cells with progesterone increases the γ2 subunit gene expression of the GABAA receptor (28). In the present study, PS increased the γ2 subunit gene expression in the hippocampus at mid and late infancy (P14 and P21), which is consistent with other studies in this context. Based on the results of existing literatures, it can be concluded that the increase of hormones related to stress and neurosteroids along with their effect on the expression of the GABAA receptor is a likely mechanism for increasing the expression of the γ2 subunit gene. On the other hand, Stone *et al*. (2001) suggested that prenatal exposure to corticosterone decreased γ2 subunit in the hippocampus (29). But, their study was different from the current study; they only applied corticosterone to the pregnant dams (not stress) and examined the pups very soon after last corticosterone injection (24 hr and 5 days); these important differences in methodology might be the reason for opposite findings.

In the present study, expression of the γ2 subunit on P21 was greater than that on P14 in both groups. This indicates the role of age in expressing this subunit in the hippocampus. It has been reported that γ2 level and other components of GABAA receptor in different parts of the brain, including the hippocampal structures, are age-specific; it has been revealed that the γ2 subunit level on the postnatal day 30 was greater than that on postnatal day 10 (30). The result of assessment of epileptic behaviors confirmed the above-mentioned studies, where significant difference was indicated between P14 and P21. In general, seizure intensity was higher on P21 compared to P14. According to this study and other similar studies, it is clear that the PS increases the susceptibility to the PTZ-induced seizure in offspring (31, 32). However, we had a contrasting finding in terms of time to onset of first seizure activity, which was longer on P21 than that of P14 in stressed pups. Since, we mentioned that seizure intensity was higher on P21 than that of P14, the longer time to onset can be a contrasting result. This result is against the study by Gholami and Saboory (2013) almost at the same age (33). We could not find a study to confirm this contrasting finding. Therefore, it might be an experimental mistake or it might have an unknown reason, which should be further elucidated.

## Conclusion

The result indicates that the prenatal stress might raise the GABAA receptor γ2 subunit gene expression in the hippocampal structures age-dependently. Furthermore, vulnerability to the PTZ-induced epileptic behaviors augmented in the prenatally stressed offspring, which might specify contribution of the GABAA receptor γ2 subunit in stress-induced potentiation of epileptic seizures in immature rats.

## References

[1] Vestergaard M, Wisborg K, Henriksen TB, Secher NJ, Ostergaard JR, Olsen J (2005). Prenatal exposure to cigarettes, alcohol, and coffee and the risk for febrile seizures. Pediatrics.

[2] Saboory E, Ebrahimi L, Roshan-Milani S, Hashemi P (2015). Interaction of prenatal stress and morphine alters prolactin and seizure in rat pups. Physiol Behav.

[3] Hashemi P, Roshan-Milani S, Saboory E, Ebrahimi L, Soltanineghad M (2016). Interactive effects of prenatal exposure to restraint stress and alcohol on pentylenetetrazol-induced seizure behaviors in rat offspring. Alcohol.

[4] Edwards HE, Dortok D, Tam J, Won D, Burnham WM (2002). Prenatal stress alters seizure thresholds and the development of kindled seizures in infant and adult rats. Horm Behav.

[5] Nejatbakhsh M, Saboory E, Bagheri M (2018). Effect of prenatal stress on a5 GABAA receptor subunit gene expression in hippocampus and pilocarpine induced seizure in rats. Int J Dev Neurosci.

[6] Maguire J, Salpekar JA (2013). Stress, seizures, and hypothalamic–pituitary–adrenal axis targets for the treatment of epilepsy. Epilepsy Behav.

[7] Joëls M (2009). Stress, the hippocampus, and epilepsy. Epilepsia.

[8] Elliott EM, Sapolsky RM (1992). Corticosterone enhances kainic acid-induced calcium elevation in cultured hippocampal neurons. J Neurochem.

[9] Olsen RW, Sieghart W (2009). GABA A receptors: subtypes provide diversity of function and pharmacology. Neuropharmacology.

[10] Ling I, Mihalik B, Etherington L-A, Kapus G, Pálvölgyi A, Gigler G (2015). A novel GABA A alpha 5 receptor inhibitor with therapeutic potential. Eur J Pharmacol.

[11] Sieghart W (2006). Structure, pharmacology, and function of GABA A receptor subtypes. Adv Pharmacol.

[12] Brockhaus J, Pape H-C (2011). Abnormalities in GABAergic synaptic transmission of intralaminar thalamic neurons in a genetic rat model of absence epilepsy. Mol Cell Neurosci.

[13] Hines RM, Davies PA, Moss SJ, Maguire J (2012). Functional regulation of GABA A receptors in nervous system pathologies. Curr Opin Neurobiol.

[14] Hirose S (2014). Mutant GABA (A) receptor subunits in genetic (idiopathic) epilepsy. Prog Brain Res.

[15] Schwarzer C, Tsunashima K, Wanzenböck C, Fuchs K, Sieghart W, Sperk G (1997). GABAA receptor subunits in the rat hippocampus II: altered distribution in kainic acid-induced temporal lobe epilepsy. Neuroscience.

[16] Gunn BG, Cunningham L, Mitchell SG, Swinny JD, Lambert JJ, Belelli D (2015). GABAA receptor-acting neurosteroids: a role in the development and regulation of the stress response. Front Neuroendocrinol.

[17] Skilbeck KJ, Johnston GA, Hinton T (2010). Stress and GABAA receptors. J Neurochem.

[18] Nakhjiri E, Saboory E, Roshan-Milani S, Rasmi Y, Khalafkhani D (2017). Effect of prenatal restraint stress and morphine co-administration on plasma vasopressin concentration and anxiety behaviors in adult rat offspring. Stress.

[19] Jin Y, Korol SV, Jin Z, Barg S, Birnir B (2013). In intact islets interstitial GABA activates GABAA receptors that generate tonic currents in α-cells. PloS one.

[20] Livak KJ, Schmittgen TD (2001). Analysis of relative gene expression data using real-time quantitative PCR and the 2− ΔΔCT method. methods.

[21] Yuan JS, Reed A, Chen F, Stewart CN (2006). Statistical analysis of real-time PCR data. BMC bioinformatics.

[22] Gholami M, Saboory E, Zare S, Roshan-Milani S, Hajizadeh-Moghaddam A (2012). The effect of dorsal hippocampal administration of nicotinic and muscarinic cholinergic ligands on pentylenetetrazol-induced generalized seizures in rats. Epilepsy Behav.

[23] Gholipoor P, Saboory E, Roshan-Milani S, Fereidoni J Effect of hyperthermia on histamine blood level and convulsive behavior in infant rats. Epilepsy Behav.

[24] Gerrow K, Triller A (2014). GABAA receptor subunit composition and competition at synapses are tuned by GABAB receptor activity. Mol Cell Neurosci.

[25] Mody I, Maguire J (2012). The reciprocal regulation of stress hormones and GABAA receptors. Front Cell Neurosci.

[26] Majewska MD, Harrison NL, Schwartz RD, Barker JL, Paul SM (1986). Steroid hormone metabolites are barbiturate-like modulators of the GABA receptor. Science.

[27] Nin MS, Ferri MK, Couto-Pereira NS, Souza MF, Azeredo LA, Agnes G (2012). The effect of intra-nucleus accumbens administration of allopregnanolone on δ and γ2 GABAA receptor subunit mRNA expression in the hippocampus and on depressive-like and grooming behaviors in rats. Pharmacol Biochem Behav.

[28] Follesa P, Serra M, Cagetti E, Pisu MG, Porta S, Floris S (2000). Allopregnanolone synthesis in cerebellar granule cells: roles in regulation of GABAA receptor expression and function during progesterone treatment and withdrawal. Mol Pharmacol.

[29] Stone DJ, Walsh JP, Sebro R, Stevens R, Pantazopolous H, Benes FM (2001). Effects of pre- and postnatal corticosterone exposure on the rat hippocampal GABA system. Hippocampus.

[30] Yu Z-Y, Wang W, Fritschy J-M, Witte OW, Redecker C (2006). Changes in neocortical and hippocampal GABAA receptor subunit distribution during brain maturation and aging. Brain Res.

[31] Gholami M, Saboory E, Roshan-Milani S (2014). Proconvulsant effects of tramadol and morphine on pentylenetetrazol-induced seizures in adult rats using different routes of administration. Epilepsy Behav.

[32] Heshmatian B, Roshan-Milani S, Saboory E (2010). Prenatal acute stress attenuated epileptiform activities in neonate mice. Yakhteh.

[33] Gholami M, Saboory E (2013). Morphine exposure induces age-dependent alterations in pentylenetetrazole-induced epileptic behaviors in prepubertal rats. Dev Psychobiol.

